# Oral Outbreak of Chagas Disease in Santa Catarina, Brazil: Experimental Evaluation of a Patient’s Strain

**DOI:** 10.1371/journal.pone.0122566

**Published:** 2015-10-15

**Authors:** Carolina S. Domingues, Daiana J. Hardoim, Celeste S. F. Souza, Flávia O. Cardoso, Verônica G. Mendes, Henrique Previtalli-Silva, Ana L. Abreu-Silva, Marcelo Pelajo-Machado, Sylvio Celso Gonçalves da Costa, Kátia S. Calabrese

**Affiliations:** 1 Laboratório de Imunomodulação e Protozoologia, Instituto Oswaldo Cruz, Rio de Janeiro, Rio de Janeiro, Brasil; 2 Centro de Desenvolvimento Tecnológico em Saúde (CDTS)/Instituto Nacional de Ciência e Tecnologia em Doenças Negligenciadas/FIOCRUZ, Rio de Janeiro, Rio de Janeiro, Brasil; 3 Departamento de Patologia, Universidade Estadual do Maranhão, São Luiz, Maranhão, Brasil; 4 Laboratório de Patologia, Instituto Oswaldo Cruz, Rio de Janeiro, Rio de Janeiro, Brasil; Federal University of São Paulo, BRAZIL

## Abstract

Chagas disease is a worldwide public health problem. Although the vectorial transmission of Chagas disease has been controlled in Brazil there are other ways of transmission, such as the ingestion of *T*. *cruzi* contaminated food, which ensures the continuation of this zoonosis. Here, we demonstrate the influence of the inoculation route on the establishment and development of the SC2005 *T*. *cruzi* strain infection in mice. Groups of Swiss mice were infected intragastrically (IG) or intraperitoneally (IP) with the *T*. *cruzi* SC2005 strain derived from an outbreak of oral Chagas disease. The results revealed that 100% of IP infected mice showed parasitemia, while just 36% of IG infected showed the presence of the parasite in blood. The parasitemia peaks were later and less intense in the IG infected mice. Mortality of the IP infected animals was more intense and earlier when compared to the IG infected mice. In the IP infected mice leucopenia occurred in the early infection followed by leucocytosis, correlating positively with the increase of the parasites. However, in the IG infected mice only an increase in monocytes was observed, which was positively correlated with the increase of the parasites. Histopathological analyses revealed a myotropic pattern of the SC2005 strain with the presence of inflammatory infiltrates and parasites in different organs of the animals infected by both routes as well as fibrosis foci and collagen redistribution. The flow cytometric analysis demonstrated a fluctuation of the T lymphocyte population in the blood, spleen and mesenteric lymph nodes of the infected animals. *T*. *cruzi* DNA associated with the presence of inflammatory infiltrates was detected by PCR in the esophagus, stomach and intestine of all infected mice. These findings are important for the understanding of the pathogenesis of *T*. *cruzi* infection by both inoculation routes.

## Introduction

Chagas disease affects more than 10 million people around the world, most of who reside in the endemic areas of 21 countries in Central and South Americas [1, 2]. According to Schimunis and Yadon in 2010 [3] Chagas disease is no longer a health problem only of Latin America becoming a worldwide health problem. Although transmission by *T*. *infestans* has been controlled in endemic countries such as Brazil, Uruguay, Chile, Venezuela and areas of Argentina and Bolivia [4, 5], there are other ways of transmission that guarantee the continuation of this zoonosis. Besides vectorial transmission, parasites may be transmitted by blood transfusion [6], congenitally [7] organ transplantation [8], laboratory accidents [9], and finally, by ingestion of contaminated food [10]. These forms of transmission are currently responsible for the introduction and maintenance of Chagas disease in non-endemic countries such as European countries, Japan, Australia, North America and the continuation of the disease in the endemic countries of Latin America [11].

Effective strategies to control vectorial transmission as well as the interruption of blood and organ transplant transmissions were adopted in several countries where the disease was endemic. Unexpectedly, other routes as oral transmission acquired importance due consumption of *T*. *cruzi* contaminated food [12].

Worldwide Brazil has the highest incidence of oral transmission. Between 2000 and 2011, 1,252 acute cases of Chagas disease were reported, and of these, 70% were attributed to oral transmission [13]. Several outbreaks of the disease from the consumption of foods and beverages contaminated with *T*. *cruzi* have emphasized the importance of this transmission route in humans [14, 15, 16, 17,18, 19, 20, 21].

Although various studies had evaluated the infectivity and the pathogenicity of intragastric infection is still required to improve understanding of the mechanisms involved in *T*. *cruzi* oral infection. Therefore, this study aims to shed further light on the pathogenesis and the influence of the inoculation route on the establishment and development of Chagas disease, by studying, in an experimental murine model, the behavior of the SC2005 strain, which was isolated from an outbreak of oral transmission in Santa Catarina, Brazil.

## Materials and Methods

### Ethics statement

All experiments with animals were performed in strict accordance with the Brazilians guidelines described in the National Council on Ethics in Research, and the protocols were approved by the Institutional Committee for Animal Ethics of FIOCRUZ (CEUA/FIOCRUZ), License Number LW16/11.

### Animals

Healthy outbred female Swiss mice, 4–6 weeks old, weighing from 20 to 22g were used. During the experiments, all mice were maintained under controlled temperature, receiving food and water *ad libitum* and were daily monitored each morning and afternoon until the end of the study.

### Parasites

#### SC2005 strain of T. cruzi trypomastigotes derived from cell culture

The SC2005 strain of *T*. *cruzi*, isolated from the peripheral blood of a man in the acute phase of Chagas disease, was used. The patient had acquired the infection orally during an outbreak of the disease in Santa Catarina, Brazil [22]. Epimastigotes of the *T*. *cruzi* SC2005 strain were maintained axenically by passages in LIT medium for 30 days, which is the period required for the occurrence of the partial metacyclogenesis forms present in the culture. Bottles of VERO cells, maintained in RPMI medium, supplemented with 10% fetal bovine serum, were infected with the metacyclic trypomastigotes. Ten days later the trypomastigotes derived from the cell culture (TCC) were obtained by washing the infected bottles and resuspending the trypomastigotes in a final volume necessary for the infection of mice.

#### SC2005 T. cruzi strain characterization

The DNA was extracted from *T*. *cruzi* epimastigotes according to Sambrook *et al*. in 1989 [23]. PCR-multiplex assay was performed using five primers; three representatives of different groups of *T*. *cruzi* strains (TCI, TCII and TcIII) were used (Table 1).

**Table 1:** PCR-multiplex assay using five primers; were used three representatives of different groups of *T*. *cruzi* strains (TCI, TCII and TcIII).

PRIMER	SEQUENCE	Tm °C
Tc1	5’-TTG CTC GCA CAC TCG GCT GCAT-3’	53
Tc2	5’-ACA CTT TCT GTG GCG CTG ATC G-3’	52
Tc3	5’-CCG CGW ACA ACC CCT MAT AAA AAT G-3’	52
Tr	5’-CCT ATT GTG ATC CCC ATC TTC G-3’	50
Exon	5’-TAC CAA TAT AGT ACA GAA ACT G-3’	42

The reaction conditions and the thermal profiles were standardized and a GeneAmp PCR System 9600 thermocycler (Perkin-Elmer) was used.

The amplification product was evaluated in 2% agarose gel using a molecular weight marker in the range of 100 base pairs.

### Experimental design

Mice were divided into three groups which were subjected to 4 hours of fasting prior to infection. Group 1 (G1): 30 animals inoculated intraperitoneally (IP) with 10^7^ TCC forms of SC2005 strain of *T*. *cruzi*, suspended in 0.2ml of RPMI medium; G2: 55 mice inoculated intragastrically (IG) with 10^7^ TCC forms SC2005 strain of *T*. *cruzi*, suspended in 0.1ml of RPMI medium; and G3 (control group): 15 normal uninfected animals. The results presented here are representative of two independent experiments.

#### Parasitemia

Five microliters of tail vein blood were put under a 22 X 22mm^2^ coverslip and the parasites were counted in 50 microscopic fields. The number of parasites/ml was estimated as described by Pizzi and Prager in 1952 [24]. Ten mice from each group were used for this procedure.

#### Mortality

Ten mice of each group were checked daily always in the morning and the mortality rate was estimated in order to obtain the surviving percentages. The mean time of death of mice was obtained based on Liddell in 1978 [25]. If any of the animals present two of the pre-established symptoms such as piloerection, partial anorexia, 10% of weight loss, vocalization, decreased mobility it was euthanized.

#### Blood leukocytes measurement

At the time of parasitemia evaluation mice were bled from the tail and samples were diluted in Turk’s solution (1/20) [26]. The cells were then counted in a hemocytometer. Differential cell counts were made on blood smears after May Grünwald–Giemsa staining by counting 100 leukocytes per slide. Measures were taken and the results are expressed either in terms of absolute numbers of cells or differential cell count.

#### Histopathology

Three animals of each group were randomly chosen and killed in accordance with the protocol approved by the Institutional Committee for Animal Ethics of FIOCRUZ (CEUA/FIOCRUZ), License Number LW16/11 on days 11 and 18 (G1—IP infected) and days 26 and 32 (G2 –IG infected) after infection, for organ recovery. The animals were anesthetized with a combination of ketamine 100 mg/kg and xylazine 2% 10mg/kg and killed with an intraperitoneal injection of Tiopental 150mg/kg, according to Brazilian legislation (CONCEA). All collected organs that were removed were fixed in 4% paraformaldehyde in PBS 0.01M, pH7.45 at 4°C for 48 hours and processed with paraffin embedding for conventional histology. Sections of 5μm thick were stained with hematoxylin eosin, Lennert’s Giemsa, Picrosirius red (Direct Red 80, Aldrich Milwaukee, WI 53233, USA) and Weigert’s resorcin-fuchsin after oxidation with oxone. The histopathological analyses were performed in a Zeiss Axioplan 2 microscope, applied to a Soft Imaging System (CC-12) camera. The inflammatory infiltrate intensity was evaluated according to Dias *et al*. [27] were 10 or more inflammatory cells per field was considered as inflammatory infiltrate and classified as follows: absent (no presence of inflammatory cells); mild (10–25 cells); moderate (26–50 cells) and intense (>50 cells).

#### Spleen and thymus index

Increases in size and weight of the spleen represent reticuloendothelial stimulation; spleen indexes were calculated after evaluation of the relative spleen weight (spleen weight/mouse weight) on days 11 and 18 (G1) and 26 and 33 (G2) after infection [28]. The same methodology was employed for thymus index.

#### PCR analysis

To detected parasite DNA in the esophagus, stomach and intestine, tissue samples of each organ were processed separately for DNA extraction according to Sambrook *et al*. in 1989 [23], and purified DNA was PCR amplified using *T*. *cruzi*-specific kDNA minicircle primers 121 (5’-AAATAATGTACGGG(T/G)GAGATGCATGA-3’) and 122 (5’-GGTTGCATTGGGTTGGTGTAATATA-3’) which amplified 330 bp fragments [29].

The reaction conditions and the thermal profile were standardized and a GeneAmp PCR System 9600 thermocycler (Applied Biosystems, Foster City, CA) was used.

The amplification product was observed in 1.5% agarose gel stained with Nancy-520 (SIGMA) using a molecular weight marker in the range of 100 base pairs.

### Statistical analysis

Results are expressed as the mean and standard error of the mean (S.E.M). Significance of total and specific leucometry as well as the spleen and thymic indexes were calculated using parametric ANOVA (one Way Analysis of Variance) and a post test Turkey-Kramer for non-parametric data. 2-Way ANOVA was used for FACs data and GraphPhad Prism for mortality evaluation.

## Results

### Parasitemia and mortality

**Fig 1:**
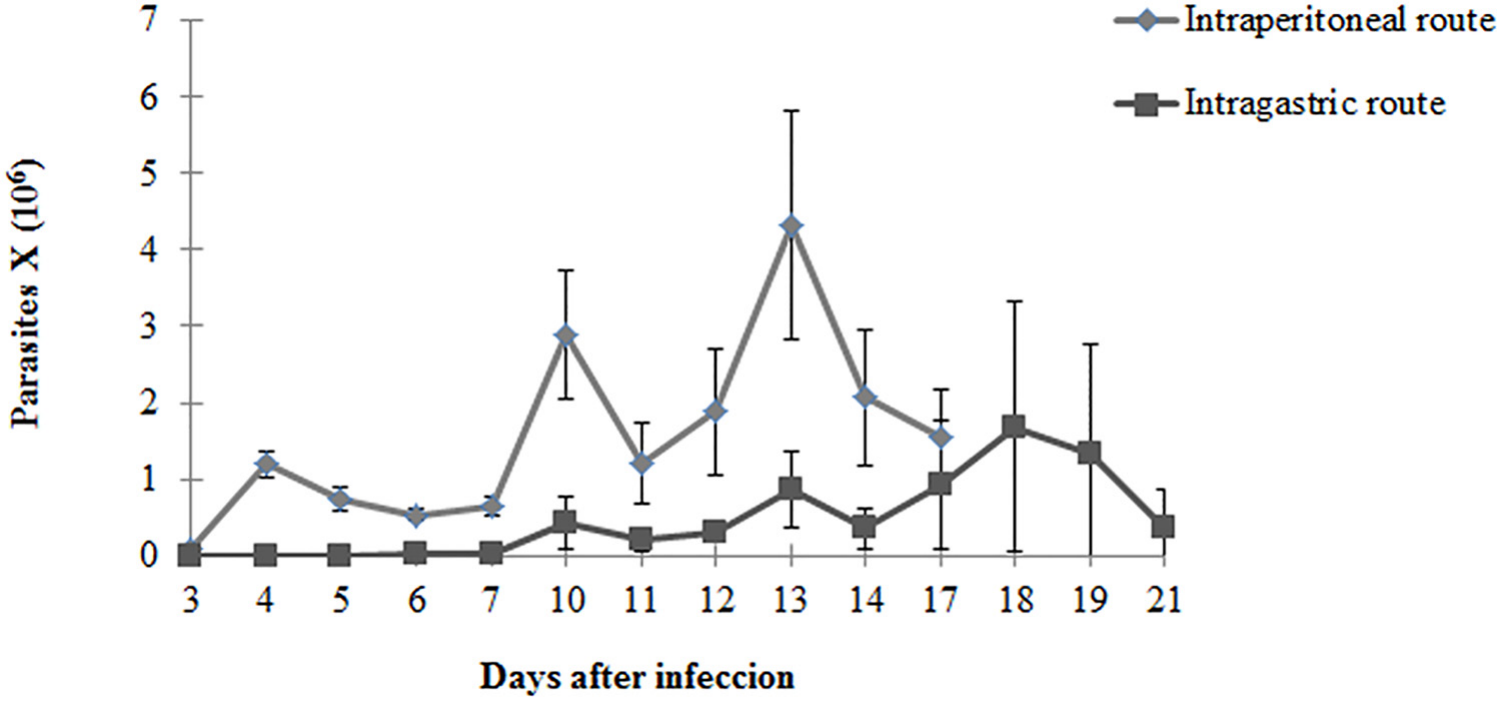
Parasitemia curve. Parasitemia curve of 4–6 week old female mice Ip or IG infected with 10^7^ culture-derived trypomastigotes of *T*. *cruzi* SC2005 strain.

Parasitemia in mice intraperitoneally infected (IP) started early on the 3^rd^ day after infection when compared with mice intragastrically infected (IG) which the presence of parasites in the blood was only observed on the 11^th^ day post infection. One hundred percent of IP infected mice showed parasitemia, while just 36% of IG infected showed the presence of the parasite in blood. As the aim of this study was to evaluate the influence of the inoculation route on the establishment and development of Chagas disease in an experimental murine model only animals that showed parasitemia were used in this study. Two peaks of parasites were observed in both IP and IG groups; however in the IP infected mice these peaks were earlier on the 10^th^ and 13^th^ days and higher (2.9 and 4.3 X 10^6^ parasites/ml respectively) than those observed in the IG infected animals which presented peaks on the13^th^ and 18^th^ days post infection with 0.9 and 1.7 X 10^6^ parasites/ml respectively (Fig 1).

Table 2 showed that IP infected animals were the first to die, and at the end of the experiment (on the 33^th^ day of infection) 80% of these animals were dead. In the IG group the mortality of animals was in the range of 30%. Mean mortality time of mice varied between 16.13±0.8 in IP and 22.67±2.0 in IG infected mice.

**Table 2:** Mortality rates and mean time of death of mice infected with 10^7^ *T*. *cruzi* trypomastigotes SC2005 strain, inoculated IP (intraperitonealy) or IG (intragastricaly).

Groups	Mortality	Mean time of death (days)
IP	(2/10)^a^	16.13±0.8^b^
IG	(7/10)^a^	22.67±2.0^b^

^a^Number survivors/Nb mice.

^b^Mean of mortality time (MMT±S.E.M.).

### Kinetics of peripheral leukocyte counts

**Fig 2:**
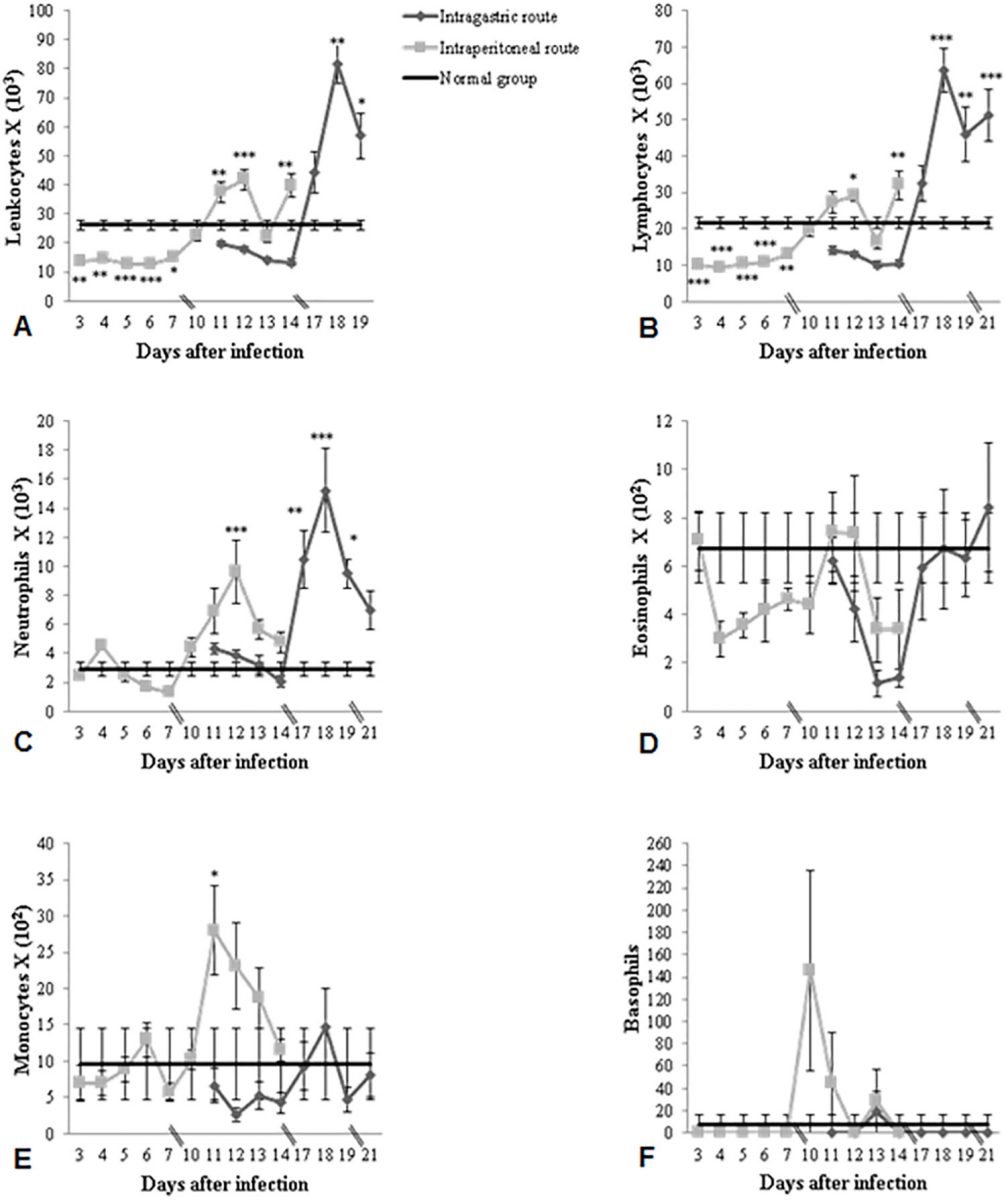
Absolute and specific number of peripheral blood leukocyte counts. Absolute and specific number of peripheral blood leukocyte counts of IP or IG infected and control mice. Mice were infected with 10^7^ trypomastigotes of *T*. *cruzi* SC2005 strain. The value for control mice is expressed by the mean of all counts.

The peripheral leukocyte cell populations of all groups were analyzed. The kinetics of total leukocyte counts of IP infected mice showed an alternate pattern of leucopenia/leucocytosis. At the beginning of the infection (from 3 up to 7 days) mice showed low amount of leukocytes. However when a parasitemia peak was observed on the 10^th^ day post infection, leukocytes reach normal values. From this point, leukocytes values reach high levels except on day 13, when a new peak of parasitemia was observed and leukocytes fell to normal values. Mice intragastrically infected showed a leucopenia pattern independent of whether the parasitemia was low or high at the beginning of the infection. However when the number of parasites began to rise on the 17^th^ day post infection a pattern of leucocytosis was observed which remained until the end of the experiment (Fig 2A).

### Differential cell counts

**Fig 3:**
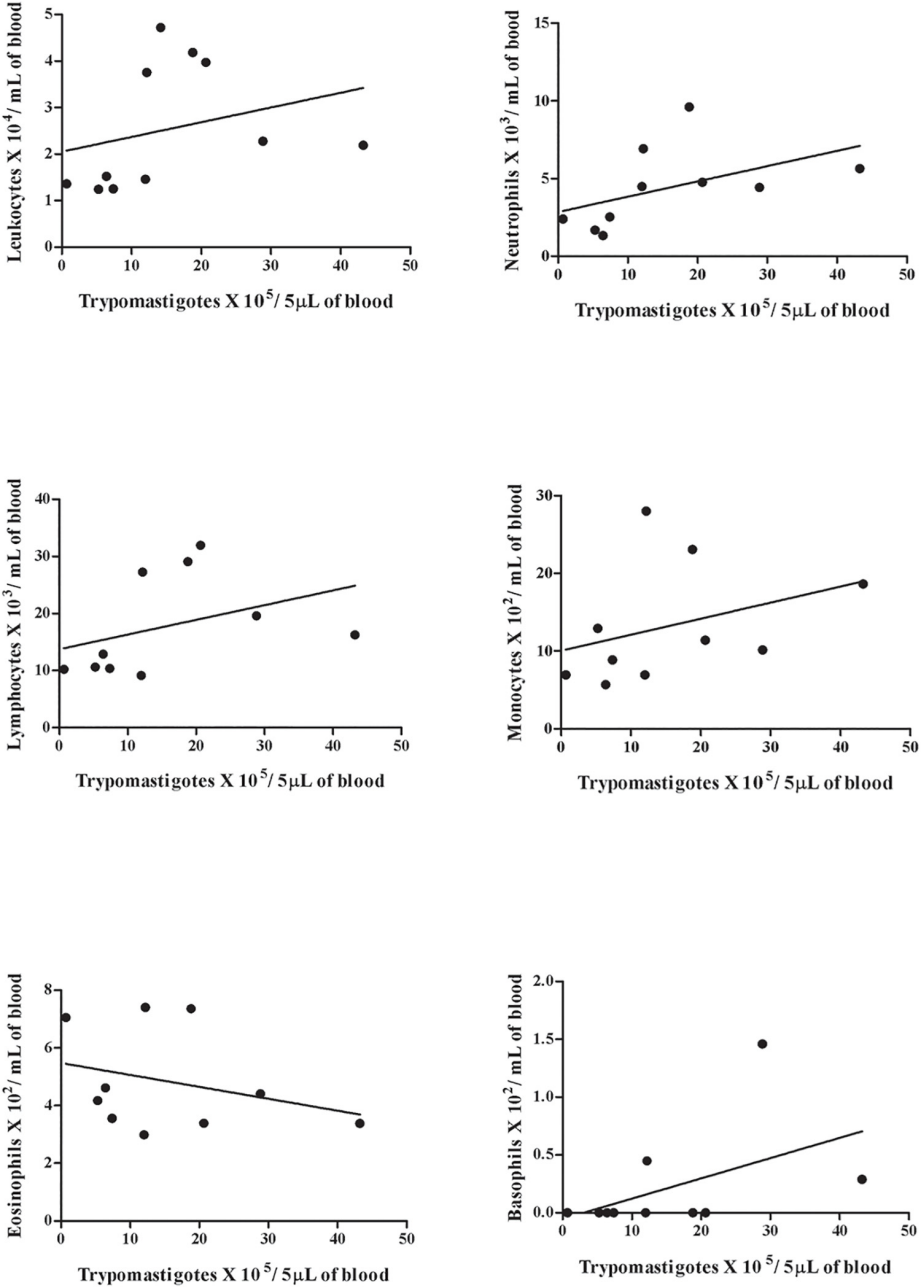
Spearman’s test of IP infected mice. Correlation between parasitemia and total leukocytes (A), neutrophils (B), lymphocytes (C), monocytes (D), eosinophils (E) and basophils (F) in animals IP infected with 10^7^ trypomastigotes forms of *T*. *cruzi*, SC2005 strain. The correlation was significant between parasitemia and white blood cell count (p = 0.023), neutrophils (p = 0.021) and lymphocytes (p = 0.042).

**Fig 4:**
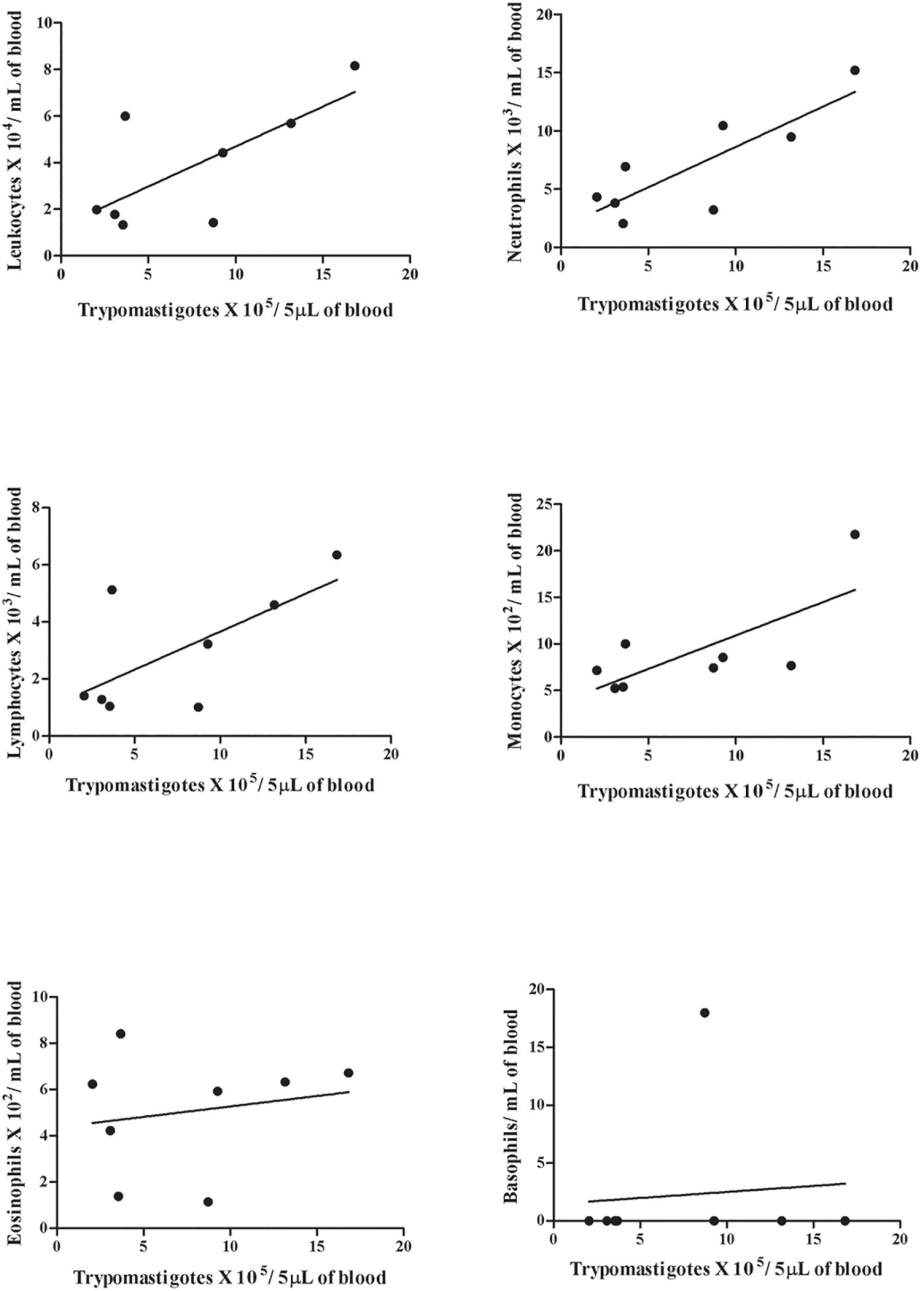
Spearman’s test of IG infected mice. Correlation between parasitemia and total leukocytes (A), neutrophils (B), lymphocytes (C), monocytes (D), eosinophils (E) and basophils (F) in IG infected animals. A significant correlation was observed between parasitemia and monocyte count (p = 0.028).

The number of monocytes, lymphocytes, neutrophils, eosinophils and basophils was counted in the blood of the Swiss mice. The counts demonstrated an increase of neutrophils, lymphocytes and monocytes and a reduction of the number of eosinophils in all infected mice, independent of the infection route. The animals infected with the SC2005 strain by IP route displayed a significant correlation of the lymphocyte and neutrophil counts with the parasitemia. There was a significant reduction in lymphocyte counts until 7 days post-infection (dpi). Lymphocytosis was observed at 12 and 14 dpi. Enhanced neutrophils counts at 12 dpi was also seen in this group. Mice inoculated with the SC2005 strain by the IG route demonstrated that the parasitemia peak (18 dpi) was correlated with the enhanced monocyte counts during the acute phase of infection. No correlation or significant alterations in basophil levels were observed in both groups. (Figs 2B–2F, 3 and 4).

### Spleen and thymus index

**Fig 5:**
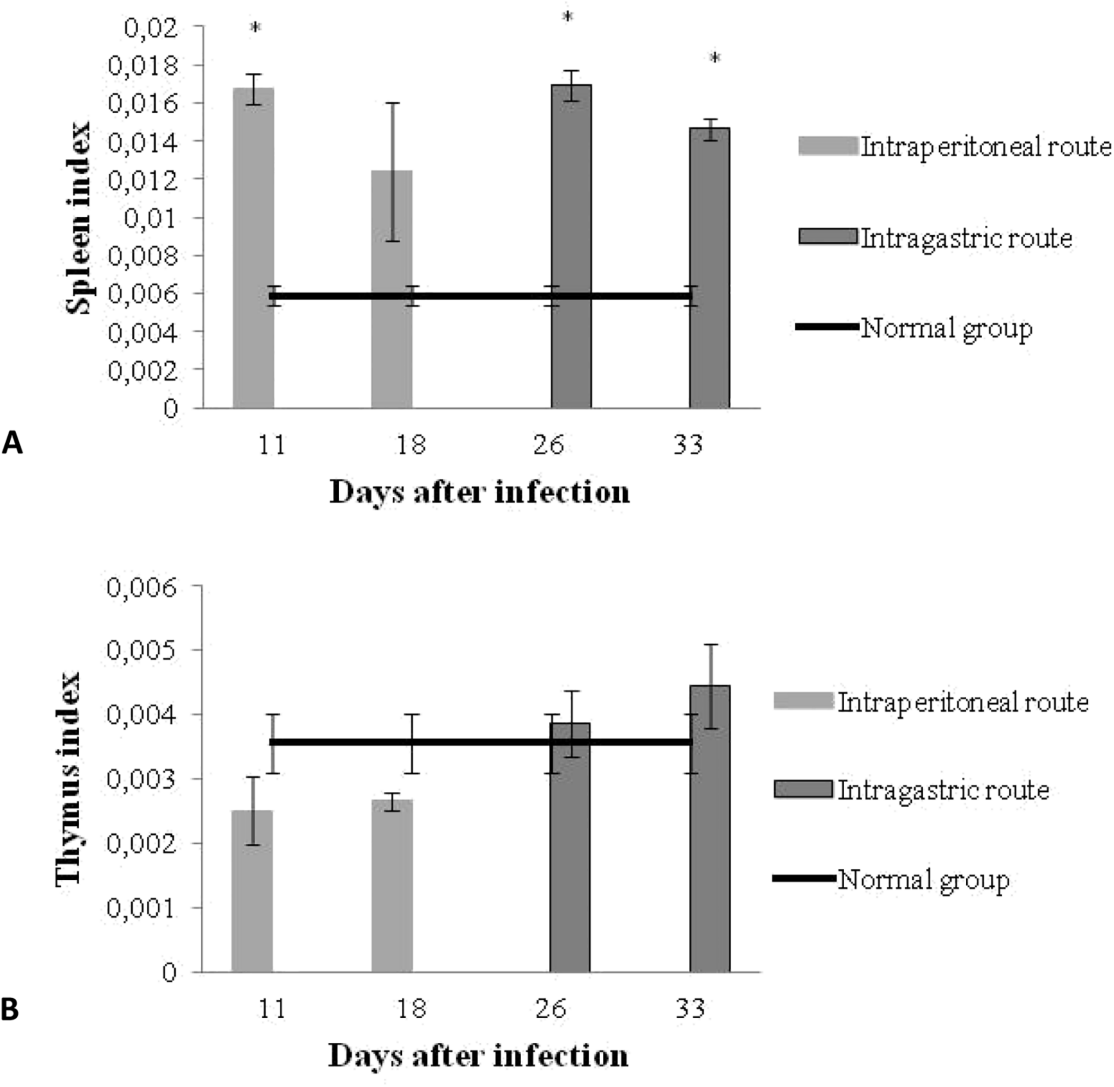
Spleen an thymus index. Spleen (A) and thymus (B) index of mice infected with 10^7^ trypomastigotes forms of *T*. *cruzi*, SC2005 strain by the IP or IG routes. The value for control mice is expressed by the mean of all counts.

The effect of infection on the spleen and thymus weight was evaluated on days 11 and 18 after infection of the IP infected mice and on days 26 and 33 post infection of the IG infected mice. The spleen reached more than twice its weight in both of the infected groups when compared to the control group (not-infected mice). When the thymus was evaluated our results showed that the *T*. *cruzi* infection by the IP route induced a reduction in the thymus index. This reduction was not observed in the IG infected mice thymus. Instead, a slight increase of this organ was observed, regardless of the infection time (Fig 5A and 5B).

### Flow cytometry

The flow cytometric analysis of the spleens from IP infected animals, 11 days after infection, showed a decrease in the number of CD8^+^ T lymphocytes and a slight increase of CD4^+^. At 18 days post-infection an increase of 50% in the number of these cells was noted when compared with non infected control mice. The analysis of the spleen from the IG infected animals, 26 six days post infection, showed approximately the same number of CD4^+^ cells observed in the control group. The number of CD8^+^ cells was 68% higher than controls and approximately 80% or 40% higher than the IP infected mice depending on the day of infection. Double positive cells of the IG infected mice increased approximately 65% when compared to the control and IP inoculated animals. At 33 days post-infection, IG infected animals showed similar profiles of CD4^+^ and CD8^+^ cells to those observed in animals at 26 days of infection. There was a reduction of double positive cells when compared to 26 days of infection, however, the number of cells was higher than those observed in the control group and in the IP infected animals after 11 days of infection (Table 3).

**Table 3:** Analysis of the T lymphocyte profiles in spleen, blood and lymph node of mice inoculated with the SC2005 strain by IP and IG routes.

			Lymphocyte subsets		
			CD4+	CD8+	CD4+/CD8+
**SPLEEN**	**Normal group**		14.97% ± 2%	6.38% ± 1%	0.18% ± 0%
	**IP Route**	**11°**	17.22% ± 2%	4.08% ± 0%	0.20% ± 0%
		**18°**	32.61% ± 0%***	12.43% ± 0%	1.10% ± 0%
	**IG Route**	**26°**	12.51% ± 1%	19.51% ± 1%***	3.05% ± 1%
		**33°**	12.29% ± 0%	20.92% ± 5%***	1.49% ± 0%
**BLOOD**	**Normal group**		29.40% ± 3%	11.43% ± 2%	1.92% ± 1%
	**IP Route**	**11°**	33.62% ± 3%	13.30% ± 5%	3.55% ± 2%
		**18°**	27.76% ± 0%	19.65% ± 0%	2.40% ± 0%
	**IG Route**	**26°**	10.38% ± 0%*	44.69% ± 0%***	3.63% ± 0%
		**33°**	18.92% ± 5%	48.11% ± 9%***	2.70% ± 0%
**LYMPH NODE**	**Normal group**		34.39% ± 3%	15.13% ± 2%	0.58% ± 0%
	**IP Route**	**11°**	25.25% ± 0%**	10.65% ± 1%	0.37% ± 0%
		**18°**	38.05% ± 0%	15.62% ± 0%	0.97% ± 0%
	**IG Route**	**26°**	20.51% ± 4%***	14.01% ± 2%	1.66% ± 1%
		**33°**	26.45% ± 1%**	15.77% ± 2%	0.62% ± 0%

*p<0.05

**p<0.01

***p<0.001.

The evaluation of the mesenteric lymph nodes from the IP infected animals after 11 days of infection showed a reduction in CD4^+^ and CD8^+^ when this mice where compared to the controls. No changes in double positive cells were observed at this point. Eight days after infection CD4^+^ and CD8^+^ cells had values similar to the control animals, although a slight increase of double positive cells was observed. In the IG infected mice there was a reduction (41%) of CD4^+^ and maintenance of CD8^+^ values after 26 days of infection. The profile of double positive cells was similar to that shown by the control group. At thirty three days of infection a reduction in the number of CD4^+^ cells was also observed, but lower than that observed at 26 days for the IG infected mice. The CD8^+^ cells showed the same profile observed in the non infected control mice, in the IP infected mice, 18 days post infection and in the IG infected mice. Double positive cells were observed in equal amounts to the control group and to both infected mouse groups (Table 3).

The analysis of the T cells in the blood of the IP infected animals, 11 days after infection, showed no change in the number of CD4^+^ and CD8^+^ cells when compared to control group. Double positive cells were shown to be nearly equal to the control group. On day 18 after infection there was no change in the number of CD4^+^ cells, however a slight increase of CD8^+^ and double positive was observed in the IP infected animals compared to the control. Twenty six days after infection IG infected animals showed a reduction of 62% of CD4^+^ and an increase of 75% in the number of CD8^+^ cells, and an increase in double-positive cells when compared to control. Thirty three days after infection the number of CD8^+^ and double positive cells was equal to that observed at 26 days for the IG infected mice. On the other hand, the amount of CD4^+^ T cells was higher in relation at IG infected animals at 26^th^ days post infection. There was a reduction in CD4^+^ cells of the IG animals at 26 and 33 dpi when compared to the control group (Table 3).

### Histopathology

#### Infection with the SC2005 strain given by the intraperitoneal route

**Fig 6:**
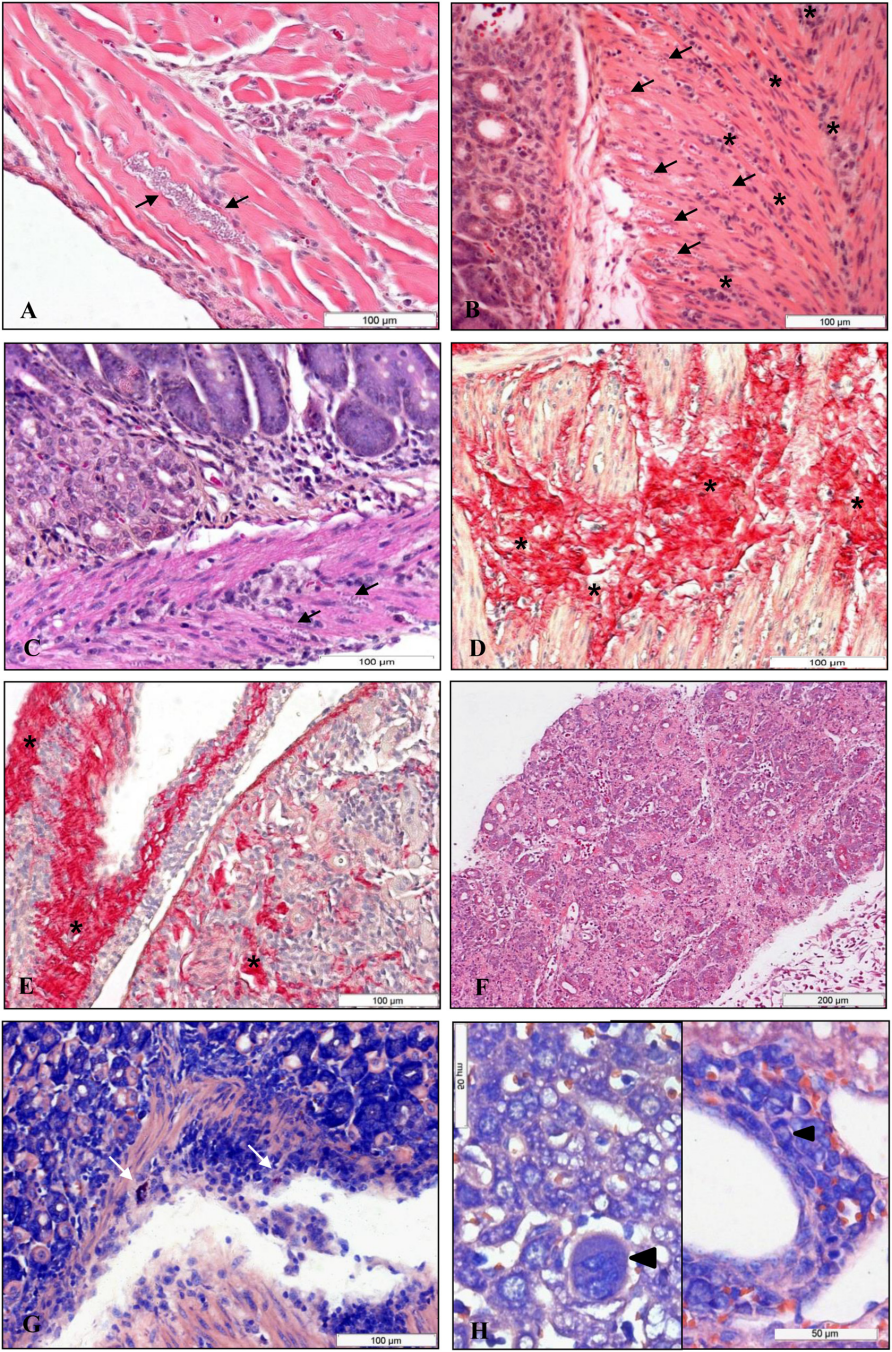
Histopathological alterations in the IP infected mice. Histopathological alterations in the mice infected IP with 10^7^ *T*. *cruzi*, SC2005 strain. (A) esophagus; (B) stomach and (C) intestine showing inflammatory cell infiltrate (asterisks) and nests of amastigotes (arrows) in the muscular layer. Hematoxylin and eosin (HE) staining. Redistribution and increase of collagen deposits (asterisks) were seen in the inflammatory foci in the stomach (D); and heart (E), Picrosirius red staining. (F) Pancreatitis with focal necrosis, HE staining. (G) Presence of mast cells (arrows) in stomach, Giemsa staining. (H) Liver showing megakaryocyte (arrow) on the left side and mitotic cells (arrow) on the right side, Giemsa stained.

To determine whether IP-given *T*. *cruzi* infection induced histopathological variations in different organs, three animals were killed on days 11 and 18 post infection and subjected to histopathological analysis. The IP infected animals showed a moderate to intense diffuse inflammatory reaction, exhibiting mainly monocytes and lymphocytes in the esophagus (Fig 6A), stomach (Fig 6B), intestine (Fig 6C), heart, liver, pancreas, adrenal gland, bladder, uterus and adipose tissue. The mucosal and submucosal layers of the different organs rarely demonstrated any mononuclear infiltration, showing normal aspects in most cases. However, in the muscle layer inflammatory infiltrates were frequently observed. Picrosirius staining showed a redistribution and increase of collagen deposits in the inflammatory foci in the esophagus, stomach (Fig 6D), heart (Fig 6E), bladder and uterus. In addition, hyperplasia of the germinal centers of the spleen and lymph nodes was seen. Mast cells were present in the adipose tissue, heart and stomach (Fig 6G). There were amastigotes in the esophagus, stomach, intestine, heart, pancreas and bladder (Fig 6A, 6B and 6C). Omental and mesenteric milky spots were activated with myeloid cells. An intense parasitism was found in the heart ventricles, however a larger number of inflammatory cells were observed in the atria. This study showed pancreatitis with focal necrosis (Fig 6F) and nests of amastigotes in the Langerhans islets. In the liver of the infected mice immature cells, eventual megakaryocytes and dividing cells were observed (Fig 6H). No significant alterations could be seen in elastic system in sections stained with Weigert’s resorcin-fuchsin after oxidation with oxone.

#### Infection with the SC2005 strain given by the intragastric route

**Fig 7:**
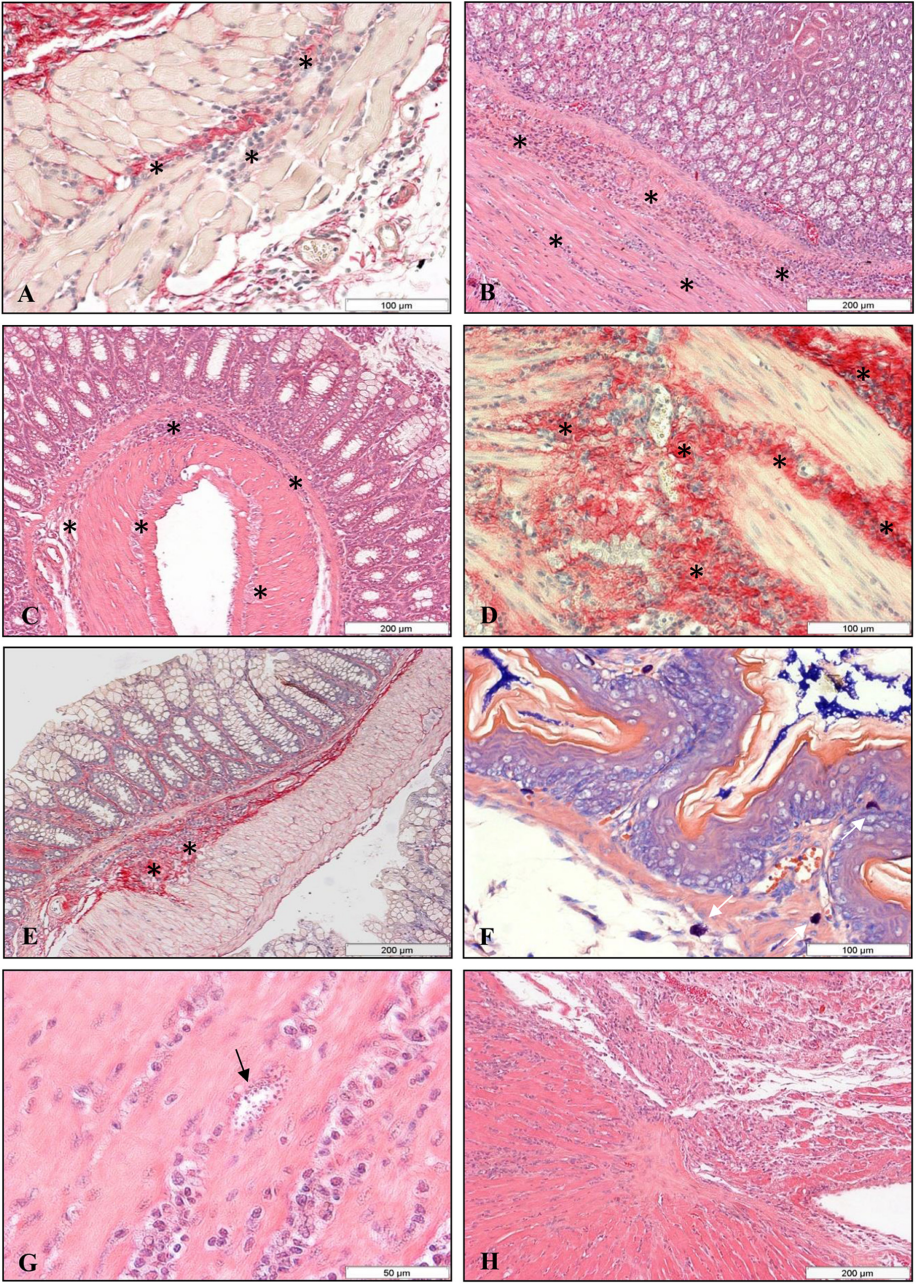
Histopathological alterations in the IG infected mice. Histopathological alterations in mice infected IG with 10^7^ *T*. *cruzi*, SC2005 strain. Inflammatory cell infiltrate in the muscular layer of esophagus (A), Picrosirius red staining; stomach (B) and intestine (C), HE staining. Redistribution and increase of collagen deposits were seen in the inflammatory foci (arrows) in the esophagus (A), stomach (D) and intestine (E), Picrosirius red staining; (F) Presence of mast cells (white arrows) in the stomach, Giemsa staining; (G) Heart showing nests of amastigotes (arrow) and (H) intense inflammatory infiltrate mainly in auricle, HE stained.

In order to verify whether IG-given *T*. *cruzi* infection caused histopathological variations in different organs three animals were killed on day 26 and 33 post infection. Sections of the organs revealed a mild to moderate diffuse mononuclear infiltration, mainly in the muscle layer of the stomach, esophagus and intestine, and in the heart, liver, pancreas, kidney, bladder, uterus, encephalon and adipose tissues (Fig 7A, 7B and 7C). Picrosirius staining showed a redistribution and increase of collagen deposits in the inflammatory foci in the esophagus (Fig 7A), stomach (Fig 7D), intestine (Fig 7E), heart, spleen, liver, pancreas, uterus and adipose tissue. Again, hyperplasia of the germinal centers of the spleen and lymph nodes was seen. Mast cells were present in the adipose tissue, bladder and stomach (Fig 7F). Parasites were scarce in the stomach, heart (Fig 7G), bladder and adipose tissue. Omental and mesenteric milky spots were activated with immature and mature myeloid cells. The lesions became more intense in the atria and ventricles of the heart of mice infected by the IG route, with fewer nests of amastigotes (Fig 7H). Immature cells and megakaryocytes were observed in the liver. No significant alterations could be seen in elastic system in sections stained with Weigert’s resorcin-fuchsin after oxidation with oxone.

#### PCR

**Fig 8:**
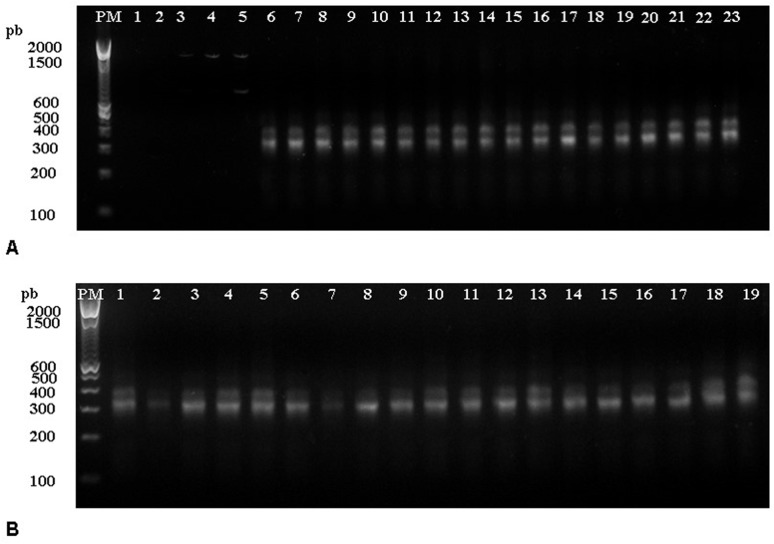
PCR. Agarose gels electrophoresis, 2%, showing the single PCR products (330pb) amplified by primers 121 and 122 extracted from different organs. (A) Non-infected animals and IP *T*. *cruzi* infected mice. Lines 1 and 2: negative control; 3 to 5: non-infected mice (control group) (3- esophagus; 4- stomach; 5- intestine); 6 to14: IP infected animals 11 dpi (6- esophagus of the mice number 1 -m1; 7- stomach (m1); 8- intestine (m1); 9- esophagus (m2); 10- stomach (m2); 11- intestine (m2); 12- esophagus (m3); 13- stomach (m3); 14- intestine (m3)); 15 to 23: IP infected mice 18 dpi. (15- esophagus (m1); 16- stomach (m1); 17- intestine (m1); 18- esophagus (m2); 19- stomach (m2); 20- intestine (m2); 21- esophagus (m3); 22- stomach (m3); 23- intestine (m3)). (B) IG *T*. *cruzi* infected mice. Lines 1–9: IG infected mice 26 dpi (1- esophagus (m1); 2- stomach (m1); 3- intestine (m1); 4- esophagous (m2); 5- stomach (m2); 6- intestine (m2); 7- esophagus (m3); 8- stomach (m3); 9- intestine (m3)); 10–18: IG infected mice 33 dpi. (10- esophagus (m1); 11- stomach (m1); 12- intestine (m1); 13- esophagus (m2); 14- stomach (m2); 15- intestine (m2); 16- esophagus (m3); 17- stomach (m3); 18- intestine (m3)); 19- positive control. PM: 100bp DNA marker ladder.

PCR was performed to detect *T*. *cruzi* DNA in the esophagus, stomach and intestine of infected mice in all points of necropsy. To check the quality of the PCR reaction, the amplification of an endogenous gene—GAPDH in the three randomly chosen samples was performed (data not shown). The samples tested showed a band corresponding to 171 base pairs, which is related to the fragment amplified for the endogen, demonstrating that the reaction proceeded satisfactorily. The PCR for parasite detection demonstrated that all samples showed a band corresponding to a fragment of 330 base pairs indicating the presence of the *T*. *cruzi* DNA in the esophagus, stomach and intestine of all infected animals (Fig 8A and 8B).

## Discussion

The transmission of the *T*. *cruzi* infection depends on several factors such as the stage or number of parasites, the routes of infection and the *T*. *cruzi* strain [30]. Little is known about the hematological, pathological and parasitological behavior of a strain isolated from the 2005 outbreak of an oral *T*. *cruzi* infection that occurred in Santa Catarina, Brazil. The findings of the present investigation indicate a similar histopathological pattern to strain TcII.

In this study, groups of outbred Swiss mice were infected intragastrically (IG) or intraperitoneally (IP) with cell culture-derived trypomastigotes of strain SC2005 as counterparts of bloodstream parasites. This form exposes on its surface a member of the gp85/trans-sialidase superfamily, Tc85-11, which is related with cell invasion and interaction with host factors. Expression of Tc85-11 on the TCC *T*. *cruzi* surface is an important condition for the parasite to translocate through the gastric mucin layer, reach the target cells and ascertain their survival within the host [31].

The results of the two different routes of infection studied here indicate a clear difference in parasitemia, mortality and infection rate. Nevertheless, inoculation by both routes produced double peaks of parasitemia in Swiss mice. An early low peak and a late high peak were observed, suggesting a mixed infection. These patterns and characteristics were similar to the TcII profiles of *T*. *cruzi* strains isolated from patients infected through oral transmission, previously described by Andrade *et al*. in 2011 [32].

Data in the literature have demonstrated that during the parasite infection molecules produced by the immune system of the host and molecules produced by *T*. *cruzi* may collaborate to produce alterations in blood cell counts [33]. Our findings indicate a positive correlation between parasitemia levels and leukocytes counts. Guedes *et al*. in 2012 [34] and Marcondes *et al*. in 2000 [35] previously reported alterations in blood cell counts associated with parasitemia levels, however different patterns of leucocytosis and leucopenia were seen. This dissimilarity may be explained by the different animals, by their heterogeneous genetic background and by different strains used in the experimental protocols.

The lymphocytosis described is concordant with the polyclonal activation of B and T lymphocytes observed elsewhere. De Meis *et al*. in 2009 [36] reported that this activation leads to increased splenic cellularity. During the *T*. *cruzi* infective process, we observed an increase in spleen weight without a loss of total body weight and a strong activation of lymphocytes in the germinal center in both IP and IG infected animals.

Our histopathological results confirmed the findings of Rassi *et al*. in 2000 [37], Opie *et al*. in 2006[38] and Castro-Sesquen *et al*. in 2013 [39] which described the presence of apoptosis and necrosis associated to the deposition of collagen in tissue remodeling during the *T*. *cruzi* infection. In the present study the histopathological analysis showed an intense mononuclear infiltrate mainly located in the muscular layers with neoformation and remodeling collagen fibers in different organs.Amastigotes were also observed in the muscular layers. These histopathological patterns were characteristic of infection with strains belonging to the biodemes type II and III [40]. In this work, we also observed an intense inflammatory lesion in the myocardium.Amastigote nests were present more frequently in the ventricles than in the atria. However, the atria were more severely affected than the ventricles, presenting an intense inflammatory infiltration with many amastigote nests, 11 days after IP infection and 26 days after IG infection. A more intense inflammatory infiltration was noted 18 and 33 days after infection in IP and IG infected mice, respectively. Increased myocardium destruction at the ventricles, less amastigote nests and more severe mononuclear cell infiltration were also noted. These findings suggest that the severe inflammatory response acts against the parasite, as it first occurred in the auricular tissue, and only reached the ventricular tissue later. It is interesting to note that Quijano-Hernández *et al*. in 2012 [41] observed the same histopathological patterns in infected dogs.

The histopathological analysis showed the presence of immature cells and megakaryocytes in the liver of all infected mice, and this cells types were observed in the lymph node of animals inoculated by the IG route at 33 days after infection. This finding suggests that extramedullary hematopoiesis was occurring in these organs. During the acute phase of *T*. *cruzi* infection, Marcondes *et al*. in 2000 [35] demonstrated alterations in blood cell counts associated with bone marrow suppression and anemia, which explain the occurrence of extramedullary hematopoiesis.

Inflammatory infiltration was found in many organs, and lymphocytes were the most frequently observed cell type. The fluctuations of the lymphocyte population observed in our study confirmed the results described by Morrot *et al*. in 2012 [42]. During the *T*. *cruzi* infection, the T-cell dynamic reflects the redistribution of lymphocyte subsets and specific and coordinated responses to the parasite in the lymphoid tissues.

The different approaches in the present study lead us to conclude that the route of infection has a direct effect on the course and the intensity of the disease. It is important to note that the SC2005 strain exhibit a specific pattern of infection that differentiates it from other strains.
